# Tumor-associated macrophages and PD-L1 in prostate cancer: a possible key to unlocking immunotherapy efficacy

**DOI:** 10.18632/aging.205378

**Published:** 2024-01-04

**Authors:** Jinhuan Wang, Wenqi Wu, Tian Yuan, Lili Wang, Li Zang, Qing Liu, Lei Wang, Xiaodong Huo, Bin Huo, Yong Tang, Haitao Wang, Zhigang Zhao

**Affiliations:** 1 Tianjin Medical University Cancer Institute and Hospital, National Clinical Research Center for Cancer, Key Laboratory of Cancer Prevention and Therapy, Tianjin's Clinical Research Center for Cancer, Tianjin 300060, China; 2 Department of Oncology, Second Hospital of Tianjin Medical University, Tianjin 300211, China; 3 Department of Medical Oncology, Tianjin First Central Hospital, School of Medicine, Nankai University, Tianjin 300192, China

**Keywords:** prostate cancer, radical prostatectomy, M2 macrophages, PD-L1, biochemical recurrence

## Abstract

Purpose: Prostate cancer (PCa) is often considered as a “cold” tumor with low responsiveness to immunotherapy. Recent evidence suggests the activation of specific immune cells, such as tumor-associated macrophages (TAMs), could potentially influence the efficacy of immunotherapy in PCa. However, the relationship between TAMs and PD-L1, a significant regulator in immunotherapy, within PCa remains unexplored.

Methods: In this study, we assessed TAM infiltration and PD-L1 expression levels in a local cohort of 95 PCa tissue samples and two publicly available PCa datasets. We employed a combination of bioinformatics and experimental techniques, including gene set enrichment analysis, CIBERSORTx, tissue microarray, immunohistochemistry staining, and analysis of single-cell sequencing datasets, to provide a comprehensive understanding of the association between PD-L1 and TAMs in the PCa microenvironment.

Results: The study showed that CD68+ TAMs and CD163+ TAMs (M2-TAMs) were more abundant in the tumor microenvironment than in non-cancerous surrounding tissues. The infiltration of CD163+ TAMs was significantly associated with the Gleason score and risk stratification of PCa. Importantly, elevated PD-L1 expression correlated significantly with high infiltration of CD163+ TAMs. Furthermore, patients displaying high levels of CD163+ TAMs and PD-L1 expression exhibited shorter times to biochemical recurrence-free survival.

Conclusion: Our study suggests that CD163+ TAMs are closely associated with PD-L1 expression and can act as a valuable prognostic indicator for PCa. The high infiltration of M2-TAMs, coupled with the overexpression of PD-L1, may contribute to immune escape mechanisms in PCa, thereby influencing disease prognosis.

## INTRODUCTION

Radical prostatectomy is widely recognized as the standard treatment for localized prostate cancer (PCa). However, over one-third of patients eventually experience biochemical recurrence (BCR) following surgery [1]. When BCR occurs, PCa often becomes highly aggressive, metastatic, and even life-threatening, particularly in cases with a high Gleason score [2]. Predicting disease recurrence or BCR is crucial for determining whether adjuvant therapy is needed. Various assessment indices, such as Gleason score, and pre-operative prostate-specific antigen (PSA) levels, are commonly used for diagnosing and prognosticating PCa [3]. Nevertheless, the appropriateness of these assessments for patients with positive surgical margins (PSM) and lymph node invasion remains debated [4]. Sensitive and specific markers for predicting cancer spread to lymph nodes at the time of diagnosis are still limited. Consequently, there is an urgent need for more effective and accurate evaluation methods to predict BCR in patients with PCa.

Tumor-associated macrophages (TAMs) are a major component of the tumor microenvironment and play a significant role in the progression of various cancers [5]. The functional polarization of TAMs and their spatial distribution have been shown to impact cancer aggressiveness, metastatic potential, and clinical outcomes for cancer patients [6–8]. TAMs play a crucial role in the intrinsic immune response, and different TAM subtypes possess distinct biological functions [9, 10]. Histologically, TAMs are divided into classically activated M1-TAMs and alternatively activated M2-TAMs, characterized by their anti-tumor and pro-tumor properties, respectively [11]. Immunohistochemistry is commonly used to identify TAMs. Antibodies against CD68, a pan-macrophage marker, allow for the identification of all macrophages regardless of their phenotype, while the CD163 marker, a transmembrane scavenger receptor for haptoglobin-hemoglobin, is highly expressed by M2 macrophages and widely recognized as an M2 macrophage marker [12, 13]. In several types of cancers, such as lymphoma, glioma, lung, gastric, thyroid, breast, and kidney cancers, high CD163 expression on TAMs has been associated with a worse prognosis. More work needs to be done concerning the clinical relevance of TAMs subpopulations in PCa. TAMs and PD-L1 have emerged as significant players in the tumor microenvironment of many cancers, including prostate cancer. TAMs, especially the M2 phenotype, have been associated with tumor promotion, suppression of T-cell-mediated immunity, and poorer prognosis in several cancers. In prostate cancer, TAM infiltration has been linked to advanced disease stages and therapeutic resistance. On the other hand, PD-L1, a ligand of the PD-1 receptor, is an immune checkpoint molecule that plays a critical role in tumor evasion from the host’s immune response. Its overexpression in tumor cells can inhibit T-cell function, leading to suppressed anti-tumor immunity. In prostate cancer, PD-L1 expression has been associated with increased aggressiveness and reduced survival rates. Considering this, the investigation of the interplay between TAMs and PD-L1 expression in prostate cancer becomes paramount. Their combined role might provide insights into disease progression, therapeutic responses, and potential avenues for targeted immunotherapies.

Immunotherapy is a hot topic in oncology treatment nowadays, and it has been well-attempted in the field of PCa. However, compared to the outstanding efficacy in lung and esophageal cancers, its application and efficacy in treating PCa appear to be limited. Clinical observations suggest that immune checkpoint inhibitor monotherapy has limited efficacy against PCa, even in tumors with high PD-L1 protein expression. Thus, additional systemic chemotherapy or targeted therapy (e.g., PARP inhibitors) may be needed, but the underlying mechanism remains to be explored [14, 15]. This work explores the prognostic significance and the relationship between TAMs and PD-L1 expression in PCa. We collected 95 resected PCa tissue samples and 3 normal prostate tissues. The protein levels of CD68, CD163, and PD-L1 were stained using specific antibodies. The association between CD274 mRNA (encoded PD-L1) and M2-TAMs, as well as the clinicopathologic characteristics of patients with PCa, was also analyzed in two independent PCa cohorts using bioinformatics-based methods. Our results may provide valuable information on TAMs and PD-L1 in the prognosis of PCa and may also guide macrophage-targeting strategies to improve the current poor efficacy of PD-1/PD-L1 blockade therapy for PCa.

## MATERIALS AND METHODS

### Patients

This retrospective study was conducted in accordance with the principles outlined in the Declaration of Helsinki and received approval from the Institutional Review Board (IRB) of the Second Hospital of Tianjin Medical University. Written informed consent was obtained from all participating patients. Our cohort included patients who underwent radical prostatectomy at the Second Hospital of Tianjin Medical University between March 2013 and December 2020. An experienced pathologist confirmed the histological diagnoses. Clinicopathological data were obtained from medical records. Formalin-fixed, paraffin-embedded tissue blocks of human tissue specimens were collected under a protocol approved by the IRB of the Second Hospital, following the principles outlined in the Declaration of Helsinki. Table 1 provides a summary of the detailed clinicopathological information for the enrolled patients.

**Table 1:** PD-L1 expression and clinical characteristics of 95 PCa patients.

**Characteristic**	**PD-L1 + (*n* = 32)**	**PD-L1 − (*n* = 63)**	***P* value**
Age (median)	64	62	
PSA (ng/mL)	9.9	8.0	**<0.01**
Gleason score (*n*, %)			**<0.01**
6	1 (3.1%)	18 (28.6%)	
3+4	10 (31.2%)	30 (47.6%)	
4+3	13 (40.6%)	7 (11.1%)	
8	5 (15.6%)	5 (7.9%)	
9	4 (12.5%)	2 (3.2%)	
Pathology(n)			0.23
Adenocarcinoma	29	62	
Ductal adenocarcinoma	1	1	
Mucinous adenocarcinoma	1	0	
Adenocarcinoma with scaly area	1	0	
Positive surgical margin (PMS, *n*, %)			**<0.01**
Yes	16 (50%)	12 (19.0%)	
No	16 (50%)	51 (81%)	
Lymph node involvement (*n*, %)			1.00
Yes	3 (9.40%)	7 (11.10%)	
No	29 (90.6%)	56 (88.9%)	
CD68 positive rate (%)	71.30	65.10	**0.04**
CD163 positive rate (%)	42.16	24.39	**<0.01**

In addition to the local cohort, this study also utilized two publicly available prostate cancer RNA-Seq datasets. Detailed information on these datasets can be found at https://portal.gdc.cancer.gov/ (TCGA-PRAD) and http://www.cbioportal.org/ (prad_su2c_2019) [16].

### Tissue microarray (TMA) construction

Three morphologically representative areas were selected from tumors of PCa patients. Furthermore, each tissue microarray (TMA) included tumor-adjacent samples (52 pairs) from the same patients who underwent radical prostatectomy, serving as internal negative controls. To verify the histopathological diagnosis and ensure adequate tissue sampling, a section from each microarray was stained with hematoxylin and eosin and then examined using bright-field microscopy.

### Immunohistochemistry (IHC) staining

The TMAs were sectioned into 3 μm slices and mounted on Flex IHC microscope slides (DakoCytomation, Glostrup, Denmark). The sections were deparaffinized in xylene and rehydrated through a graded alcohol series. Antigen retrieval was performed by heating the sections with Envision Flex Target Retrieval solution at high pH (Dako, Glostrup, Denmark). Staining was conducted at room temperature using an automatic staining workstation (Dako Autostainer Plus, Dako). The primary antibodies used included rabbit anti-PD-L1 antibodies (1:100 dilution, ab205921, Abcam, Cambridge, UK), rabbit anti-CD68 antibodies (1:50 dilution, BA3638, Boster, California, UK), and rabbit anti-CD163 antibodies (1:200 dilution, bs-2527R, Bioss, Beijing, China). Negative controls were prepared by omitting the primary antibody. Four observers, blinded to clinical data, independently evaluated the IHC results. The number of CD163+ cells was counted in each 1 mm² area from three independent high-power representative microscopic fields (HPFs, 400×; 0.0625 μm²), in both the tumor nests and surrounding stroma. For survival analysis, all samples were divided into low and high groups based on the number of positive cells/mm², using cut-off values of 50 (median) for both CD68+ and CD163+ cells. PD-L1 expression in more than 10% of tumor cells was associated with poorer survival [17], which was established as a cut-off point for subsequent analyses. Specimens were classified into two categories based on tumor cell proportion score (TPS): negative (<10%) and positive (10%–100%).

### Publicly available datasets analysis

In this study, two independent RNA-Seq datasets were utilized. The TCGA-PRAD (prostate adenocarcinoma) dataset’s RNA-Seq FPKM data was obtained from The Cancer Genome Atlas (TCGA) database (https://portal.gdc.cancer.gov/). Batch effect analysis was conducted using TCGA Batch Viewer (https://bioinformatics.mdanderson.org/public-software/), and no significant batch effect was observed. Clinical follow-up information, as well as pathological information, was downloaded from the Xena database (http://xena.ucsc.edu/). For the prad_su2c_2019 dataset, both mRNA expression data and clinicopathological information were obtained from the cBioPortal database (http://www.cbioportal.org/). Clinical information was manually reviewed, and cases lacking essential information were excluded from the study. Detailed clinicopathologic information can be found in Supplementary Tables 1 and 2.

### Gene set enrichment analysis

To investigate the hallmarks and pathways enriched in the predicted high- and low-risk groups, Gene Set Enrichment Analysis (GSEA) was performed as previously described [18]. The infiltration level of M2 macrophages was used as the ranking metric to generate the ranked gene list in the two RNA-Seq datasets. GSEA was conducted using the WebGestalt online program (http://www.webgestalt.org/), with the WikiPathway gene set employed in the analysis.

### Immune infiltration analysis by CIBERSORTx

The immune infiltration levels of 22 immune cells were determined using the CIBERSORTx program (https://cibersortx.stanford.edu/) with bulk-tumor RNA-Seq data as input. CIBERSORTx was executed in both relative and absolute models, incorporating S-mode batch effect correction. The RNA-Seq FPKM data and the built-in LM22 signature matrix were used as input.

### Singe cell RNA-seq data analysis

The single-cell RNA-Seq data were analyzed using the TISCH2 database (http://tisch.comp-genomics.org/search-gene/). Six single-cell RNA-Seq datasets were selected for this study: GSE137829 (Single-cell analysis supports a luminal-neuroendocrine transdifferentiation in human prostate cancer), GSE141445 (Single-cell analysis reveals the onset of multiple progression-associated transcriptomic remodellings in prostate cancer), GSE143791 (Human prostate cancer bone metastases have an actionable immunosuppressive microenvironment), GSE150692 (Single-cell RNA-seq analysis of wild-type mouse and benign human prostate), GSE172301 (Single-cell RNA-sequencing of adult human prostates from BPH patients), and GSE176031 (Single-cell analysis of human primary prostate cancer reveals the heterogeneity of tumor-associated epithelial cell states) [18–23]. The data were presented using UMAP (Uniform Manifold Approximation and Projection) for the visualization of cell clusters [19–24].

### Statistical analysis

All statistical analyses were conducted using SPSS version 18 (IBM Co., Armonk, NY, USA) or R software (version 3.6.0). Chi-square tests and Fisher’s exact tests were utilized for comparisons between categorical variables. Mean value comparisons were performed using *t*-tests. BCR-free time was defined as a PSA ≥0.2 ng/mL followed by a subsequent confirmatory value of ≥0.2 ng/mL. Survival curves were plotted using the Kaplan-Meier method. Differences in survival times between patient subgroups were compared using Mantel’s log-rank test. GEPIA (Gene Expression Profiling Interactive Analysis, http://gepia2.cancer-pku.cn/#index) online tools were used to determine the differentially expressed genes in the TCGA database. All *P* values were based on two-sided statistical analysis, and a *P* value less than 0.05 was considered statistically significant.

### Availability of data and materials

All datasets used in this study are publicly available from the corresponding database or provided as Supplementary Materials.

## RESULTS

### Expression patterns of CD68 and CD163 proteins in PCa

**Figure 1:**
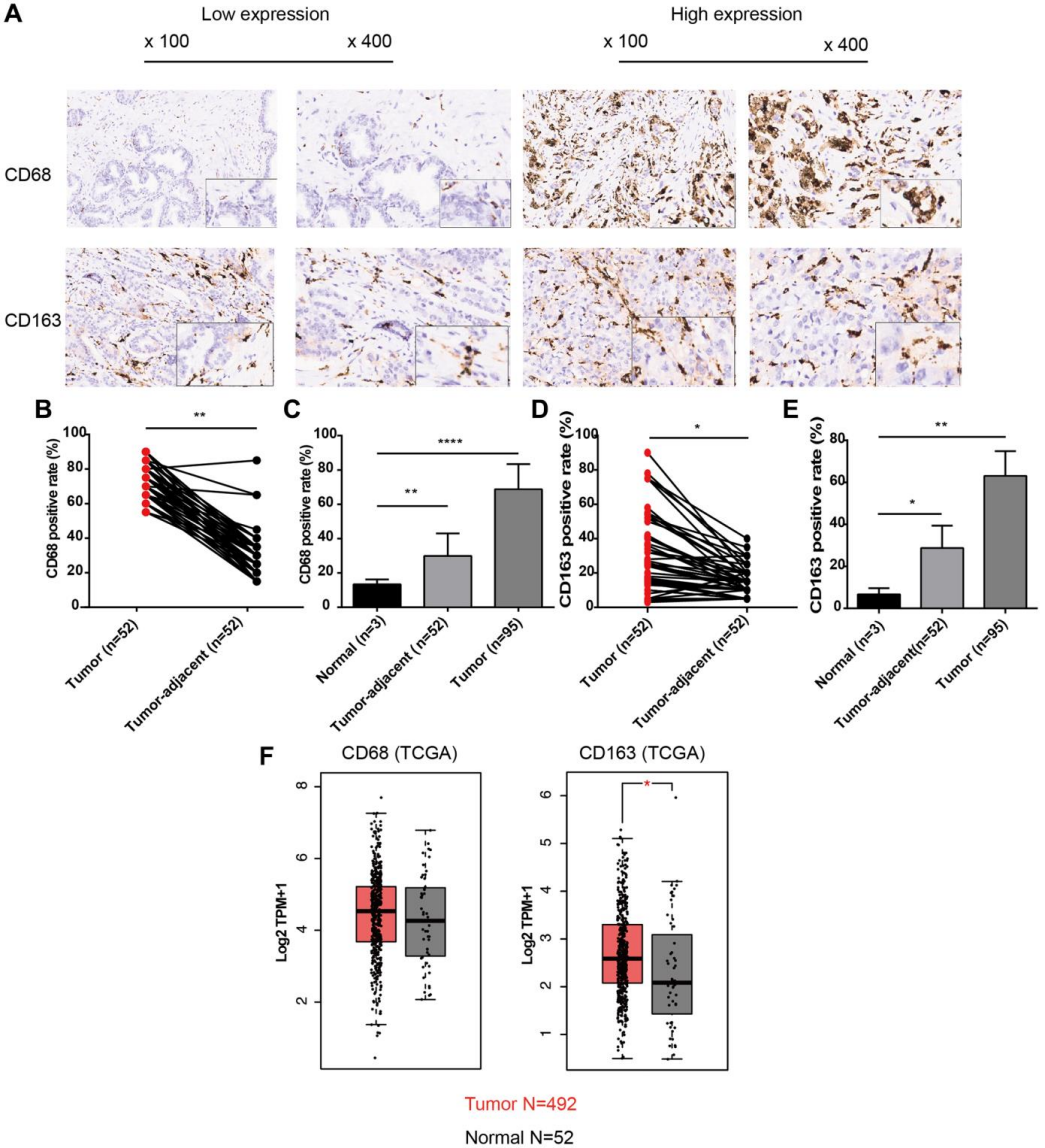
**Expression patterns of CD68 and CD163 proteins in PCa and normal prostate tissue.** (**A**) Representative IHC images of PCa tissue slides with low (left panel) or high (right panel) levels of CD68 and CD163 protein. IHC, immunocytochemistry. (**B**) Dot plot shows the positive rate of CD68 in PCa and para-cancer (para-PCa) tissue. (**C**) Bar plot shows the positive rate of CD68 in normal prostate tissue, para-PCa, and PCa. (**D**) Dot plot shows the positive rate of CD163 in PCa and para-PCa tissue. (**E**) Bar plot shows the positive rate of CD163 in normal prostate tissue, para-PCa, and PCa. (**F**) Box plots showed the mRNA expression levels of CD68 and CD163 in the TCGA-PRAD dataset. Student’s *t*-test, ^*^P < 0.05, ^**^P < 0.01, ^****^P < 0.001.

To analyze the infiltration levels of TAMs in PCa, we first observed the general expression patterns of CD68 (TAM-marker) and CD163 (M2-TAM marker) proteins in PCa and adjacent normal tissues using TMAs containing 96 PCa samples (52 with matched adjacent normal tissues). The IHC results demonstrated that CD68 and CD163 in PCa tissues were diffusely expressed in stromal cells with membranous and cytoplasmic staining, indicating that TAMs were indeed infiltrating PCa tissues with a dispersed distribution pattern (Figure 1A). Moreover, tumor cells exhibited less positive staining with CD68 and CD163. We further analyzed the signal intensity of CD68 and CD163 proteins in cancerous and normal tissues, revealing that the positive rate of CD68 in PCa was significantly higher than that in tumor-adjacent and normal tissues, with average percentages of 68.68 ± 14.72, 29.90 ± 13.15, and 13.33 ± 2.89, respectively (Figure 1B, 1C, *P* < 0.01). These findings were similar to the results for CD163 (Figure 1D, 1E, 63.07 ± 11.76 vs. 28.75 ± 10.61 vs. 6.67 ± 2.89, *P* < 0.05). In addition to the IHC results, CD68 and CD163 were also upregulated at the mRNA level in TCGA-PRAD dataset (Figure 1F). These results suggested that the infiltration levels of TAMs, including CD68+ TAMs and CD163+ TAMs (M2), were increased in PCa.

### The infiltration levels of M2 TAMs were positively correlated with Gleason score

**Figure 2:**
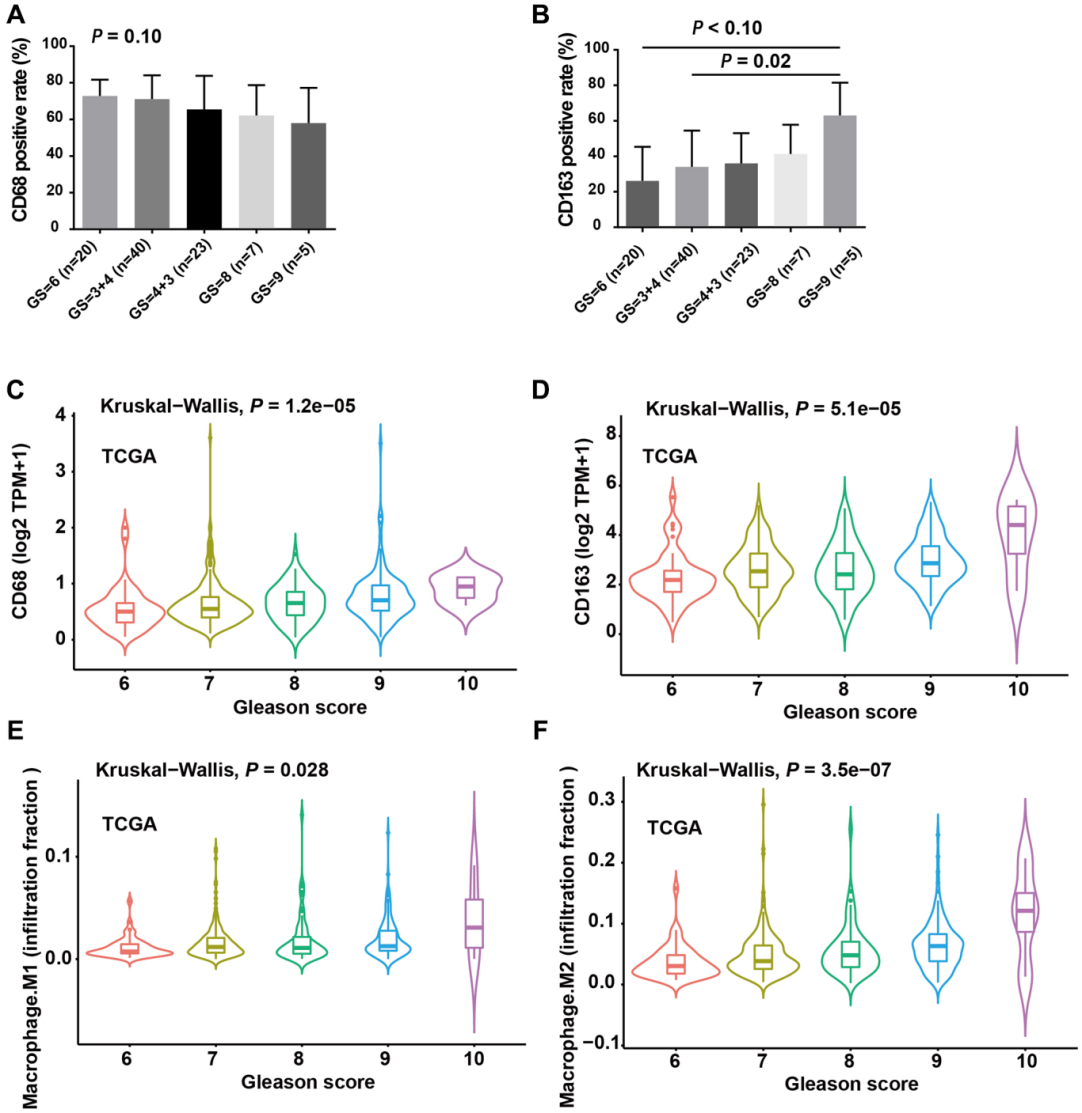
**The infiltration levels of M2 TAMs were positively correlated with Gleason score.** (**A**) CD68 positive rate was moderately negatively correlated with Gleason score (GS). (**B**) CD163 positive rate increased with increasing GS and the difference was more obverse between GS = 9 and GS = 6/3+4 group. (**C**) Violin plot showed the expression level of CD68 mRNA with increasing GS. (**D**) The expression level of CD274 mRNA was positively correlated with GS. (**E**) Violin plot showed the infiltration levels of M1 macrophages with increasing GS. (**F**) Violin plot showed the infiltration levels of M2 macrophages with increasing GS. A, B and C. *ANOVA* test; C, D and E, Kruskal-Wallis test.

To investigate the clinical significance of infiltrating TAMs in patients, we first analyzed the IHC signals of CD68 and CD163 in PCa tissue, along with detailed clinicopathological information. The results revealed that CD68 had no significant associations with Gleason score (Figure 2A, *P* = 0.10), whereas the level of CD163 protein was significantly elevated in PCa tissues with a high Gleason score (Figure 2B, Gleason score = 7 vs. Gleason score = 9, *P* = 0.02). To further support our findings, we analyzed CD68 and CD163 mRNA expression in the TCGA prostate cancer dataset (TCGA-PRAD), which contains 495 PCa samples. Consistent with our local cohort, a high expression of CD163 mRNA was significantly associated with an advanced Gleason score (Figure 2C); although CD68 mRNA expression also increased with Gleason score, it was less statistically significant compared to that of CD163 (Figure 2D). Additionally, we calculated the infiltration levels of M2- and M1-TAMs in the TCGA-PRAD dataset using the CIBERSORTx program. Correlation analysis indicated that the infiltration level of M2-TAMs was significantly elevated in PCa with a higher Gleason score (Figure 2F, *P* = 3.5e-07), while the infiltration level of M1-TAMs showed a weaker association with Gleason score (Figure 2E, *P* = 0.026). Collectively, these results suggested that the infiltration of CD163+ TAMs (M2) is closely linked with the risk stratification of PCa. Both our local cohort and the TCGA-PRAD dataset indicated more pronounced CD163 expression and M2-TAM infiltration in PCa with highly aggressive features.

### M2-TAMs infiltration was positively associated with PD-L1 expression

**Figure 3:**
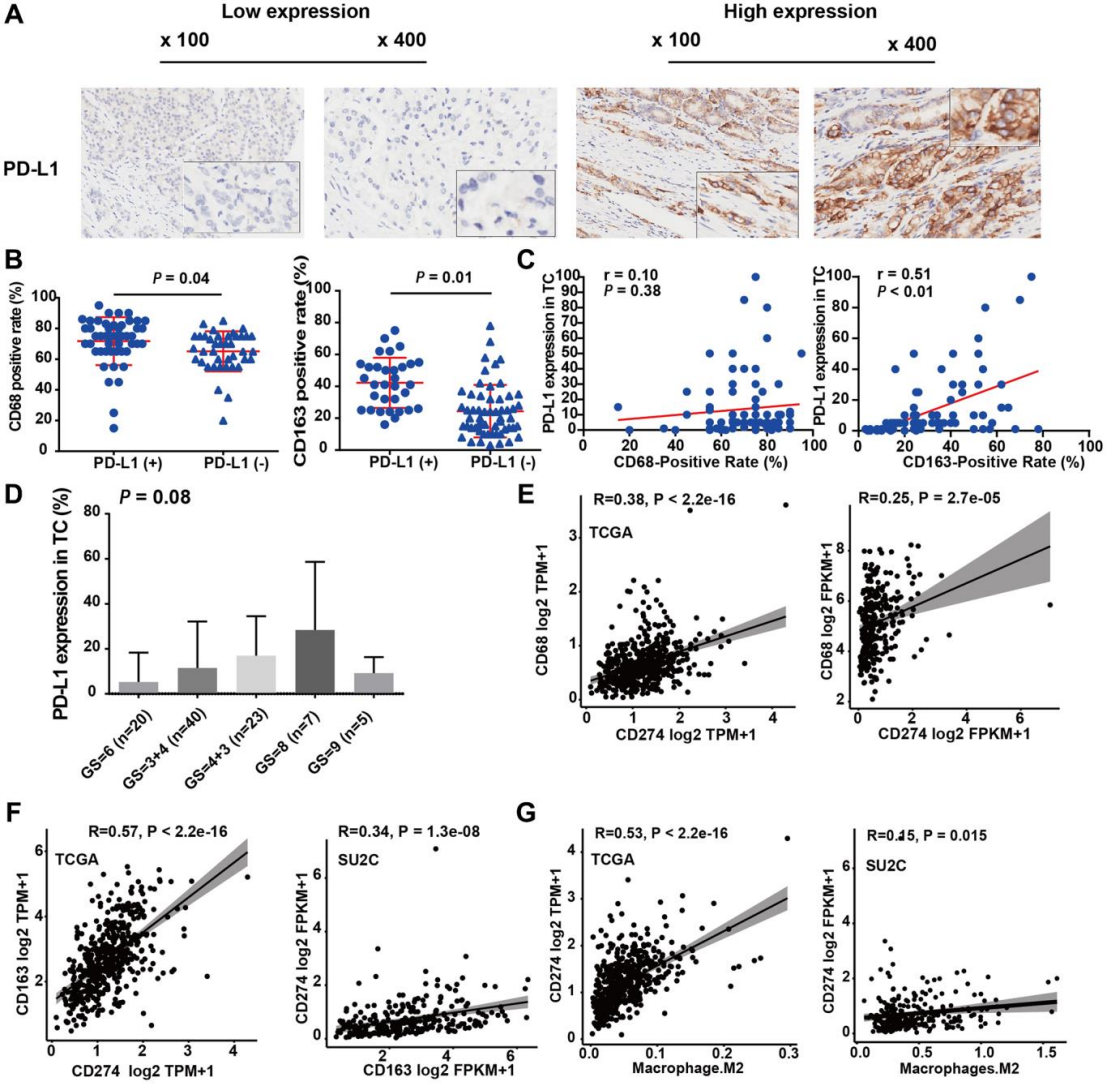
**M2-TAMs infiltration was positively associated with PD-L1 expression.** (**A**) Representative IHC images of PCa tissue slides with low (left panel) or high (right panel) levels of PD-L1 protein. (**B**) Dot plot shows the expression of CD68 in PD-L1 (+) and PD-L1 (−) PCa (Student’s *t*-test). (**C**) Dot plot shows the expression of CD163 in PD-L1 (+) and PD-L1 (−) PCa (Student’s *t*-test). (**D**) Bar plot showed PD-L1 protein expression increasing with Gleason score (ANOVA test). (**E**) Correlation between CD274 mRNA (encoding PD-L1) and CD68 mRNA in TCGA-PRAD and prad_su2c_2019 dataset. (**F**) Correlation between CD274 mRNA and CD163 mRNA in the two independent PCa datasets. (**G**) Correlation between the infiltration levels of M2 macrophages and the expression levels of CD274 mRNA in the two independent PCa datasets.

We further investigated the association between TAMs and PD-L1, a key regulator associated with the efficacy of immune-blockade therapy. IHC results demonstrated that in 14.4% (18 out of 96) of PCa samples, more than 10% of tumor cells showed a positive PD-L1 IHC signal, which is consistent with previous reports [25]. These data indicated that PD-L1 expression levels were mainly low to moderate for PCa patients (Figure 3A). The staining levels of CD68 and CD163 in the PD-L1+ group were higher than those in the PD-L1− group (CD68: 72.11 vs. 66.36, *P* = 0.13; CD163: 41.83 vs. 28.03, *P* < 0.01; Table 1 and Figure 3B). Additionally, the expression levels of CD163 and PD-L1 were positively correlated (r = 0.51, *P* < 0.01; Figure 3B right panel). However, no significant correlation was found between the expression levels of CD68 and PD-L1 (r = 0.10, *P* = 0.38, Figure 3C left panel).

Moreover, IHC also showed that PD-L1 protein expression was positively correlated with the Gleason score of PCa patients (Figure 3D). In addition, we analyzed the differentially expressed genes in samples with high and low PD-L1 expression. We found that among these, 713 genes were upregulated and 83 genes were downregulated (Supplementary Figure 1A, 1B). This result suggests that the genes positively correlated with PD-L1 might play a more significant biological role. We performed an enrichment analysis on these differentially expressed genes using both the HALLMARKS 50 gene set (Supplementary Figure 1C, 1D) and the KEGG gene set. The results revealed that several immune-related pathways, such as “interferon gamma”, “interferon alpha”, “Cytokine-cytokine receptor interaction”, and “Chemokine signaling pathway”, were significantly enriched. However, for genes negatively correlated with PD-L1, we used GSEA for analysis (Supplementary Figure 1E). We found pathways like oxidative phosphorylation, MYC target genes, and DNA repair to be enriched, but the statistical significance was not very pronounced. These findings suggest that the elevated expression level of PD-L1 in PCa is closely related to immune response and activation, consistent with the classic function of PD-L1.

We further analyzed the mRNA expression correlation among CD68, CD163, and CD274 (encoding the PD-L1 protein) in two independent datasets, TCGA-PRAD, and prad_su2c_2019. In line with our local cohort, the results demonstrated that CD274 mRNA expression was significantly positively correlated with CD68 (Figure 3E) and CD163 (Figure 3F) mRNA expression. Notably, the infiltration level of M2-TAMs was also strongly correlated with CD274 expression in TCGA-PRAD and prad_su2c_2019 cohorts (Figure 3G). This is in agreement with our IHC data, which indicated that PCa with higher levels of M2-TAM infiltration exhibited elevated levels of PD-L1 expression. Furthermore, CD163 mRNA expression was also significantly positively associated with CD68 mRNA expression in the two PCa RNA-Seq cohorts (Supplementary Figure 2A). Additionally, CD68 mRNA and CD163 mRNA expression were positively associated with the levels of M2-TAM infiltration (Supplementary Figure 2B, 2C). In summary, these results suggested that PD-L1 expression is associated with M2-TAM infiltration in PCa.

### M2-TAMs infiltration and PD-L1 expression are associated with the prognosis of PCa patients

**Figure 4:**
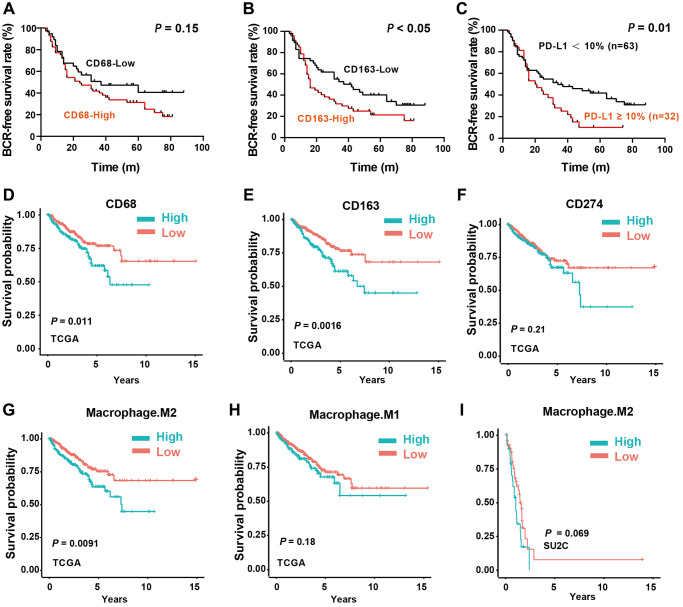
**Impact of M2-TAMs Infiltration and PD-L1 expression on the prognosis of PCa patients.** (**A**–**C**) Kaplan-Meier analysis of the protein expression levels of CD68, CD163, and PD-L1 with biochemical recurrent (BCR) free survival time. (**D**–**F**) Kaplan-Meier analysis of the mRNA expression levels of CD68, CD163, and CD274 with BCR free survival time in TCGA-PRAD dataset. (**G**, **H**) Kaplan-Meier analysis of the infiltration levels of M1- and M2- TAMs with progress-free survival (PFS) in TCGA-PRAD dataset. (**I**) Kaplan-Meier analysis of the infiltration levels of M2-TAMs with overall survival (OS) in prad_su2c_2019 dataset.

To further address the clinical significance of M2-TAMs and PD-L1 in PCa patients, we first analyzed the association of TAMs markers CD68 and CD163 with BCR-free time in our local cohort. The median follow-up time was 86 months (m), 68 (71.6%) patients experienced BCR after radical prostatectomy, and the median BCR-free time was 33.03 m (3 m – 88 m). The rates of 1-, 2-, and 5-year BCR-free survival rates were 78%, 53.4%, and 31%, respectively. No statistical significance was found in the BCR-free time of patients between low- and high-CD68 expression groups (*P* = 0.15, Figure 4A). As for CD163, the BCR-free time of the patients with low CD163 expression was significantly longer than that of the patients with high CD163 expression (mean BCR-free time: 45.6 m vs. 31.8 m, *P* < 0.05, Figure 4B). Notably, the BCR-free time of the high PD-L1 expression group was shorter than that of the PD-L1 low expression group (median BCR-free time: 24.67 m vs. 35.3 m, *P* = 0.01, Figure 4C). These results indicated that increased CD163+ TAM (M2) infiltration and high PD-L1 expression were correlated with shorter BCR-free time and could be recognized as poor prognostic factors for PCa patients. In addition, in COX regression analysis models, the CD163 IHC signal was an independent adverse factor (HR = 2.48; *P* = 0.04), indicating that M2-TAMs could independently predict the prognosis of patients with PCa (Table 2).

**Table 2:** Univariate analysis of Biochemical Recurrence (BCR)-free survival.

**Clinical or Pathologic Features**	**BCR-Free time**	
	**Hazard Ratio (95% CI)**	***P* value**
PD-L1 expression (tumor cells)		
Negative	1 (reference)	
Positive	2.66 (1.21–5.85)	0.09
CD68-positive cell density		
Low	1 (reference)	
High	0.88 (0.41–1.89)	0.31
CD163-positive cell density		
Low	1 (reference)	
High	2.48 (1.12–5.50)	0.04

In support, we also analyzed the clinical significance of CD68 and CD163 mRNA expression in TCGA-PRAD and prad_su2c_2019 cohorts. Consistent with the local cohort, high CD68 (Figure 4D) and CD163 (Figure 4E) mRNA expression were associated with poor prognosis of PCa patients. However, CD274 mRNA expression showed no association with the prognosis, which is also consistent with the IHC data (Figure 4F). Notably, we found that the infiltrated M2-TAMs had significant prognosis value in predicting DFS (Figure 4G), whereas M1-TAMs were not correlated with the clinical outcome of PCa patients (Figure 4H). Consistently, the infiltration level of M2-TAMs was correlated with the prognosis in the SU2C dataset (Figure 4I). In summary, these results suggested that PD-L1 protein expression is a promising biomarker for outcome prediction, and M2-TAMs infiltration is tightly linked with the progression of PCa.

### M2-TAMs infiltration is a key factor involved in immune modulation in PCa

**Figure 5:**
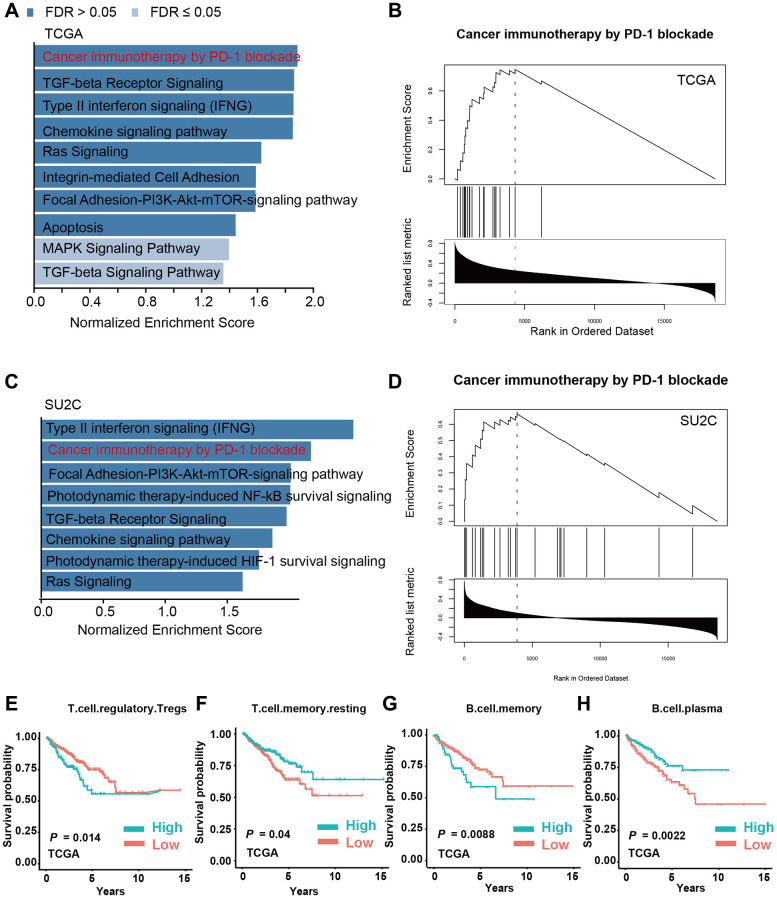
**M2 macrophage infiltration is a key factor involved in immune modulation in PCa.** (**A**, **B**) Gene set enrichment analysis (GSEA) of PCa patients with high and low infiltration levels of M2 macrophages in the TCGA-PRAD dataset. (**C**, **D**) GSEA analysis of PCa patients with high and low infiltration levels of M2-TAMs in prad_su2c_2019 dataset. (**E**–**H**) Kaplan-Meier analysis of PCa patients with high or low infiltration levels of T regulatory cells, T memory cells, B memory cells, and B plasma cells with PFS in TCGA-PRAD dataset.

Since we have identified that M2-TAMs infiltration is tightly linked with the prognosis of PCa, we investigated the corresponding molecular foundations. We performed GSEA using the infiltration levels of M2-TAMs as the metric in two PCa data sets, TCGA-PRAD and prad_su2c_2019 against the WikiPathway geneset. The results showed that PD-1 blockade-related genes were highly expressed in PCa samples with a high level of M2-TAMs infiltration in the two analyzed datasets, which further supports the intrinsic connections between M2 macrophages and PD-1-related immune therapy (Figure 5A–5D). We next analyzed the survival-prediction potential of the additional 22 immune cells using the CIBERSORTx-LM22 signature matrix. The results showed that, in addition to M2-TAMs, 4 immune cell types, including regulatory T cells, memory T cells, memory B cells, and plasma cells, also showed a strong association with prognosis (Figure 5E–5H).

**Figure 6:**
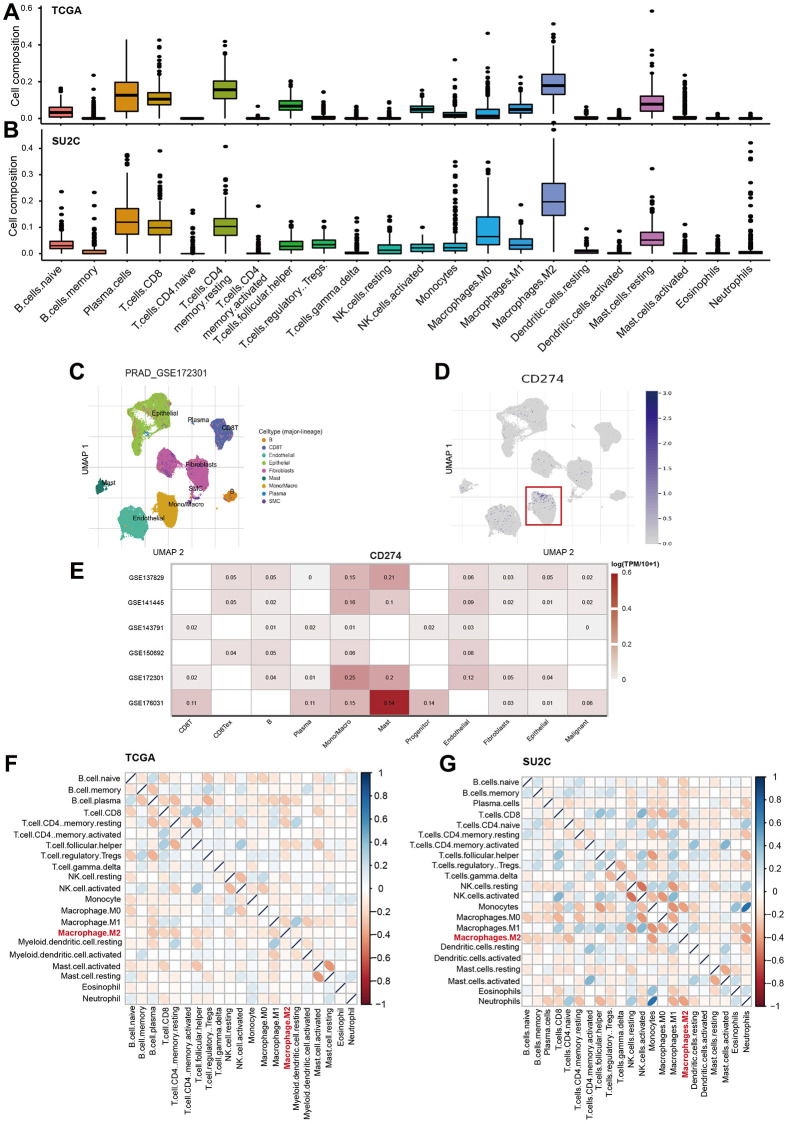
**M2-TAMs are major immune-modulator in PCa.** (**A**, **B**) The landscape of 22 immune cells in PCa. The infiltration factions of the 22 analyzed cell types were calculated by CIBERSORTx program in TCGA-PRAD (**A**) and prad_su2c_2019 datasets (**B**). (**C**, **D**) UMAP showed the distribution of cell clusters in the GSE172301 dataset, and the expression levels of CD274 mRNA were shown as blue dots in log2TMP+1. (**E**) Heatmap showed the expression levels of CD274 mRNA across multiple cell types in TME. (**F**, **G**) Heatmap showed the correlation among the 22 immune cells in TCGA-PRAD (**F**) and prad_su2c_2019 dataset (**G**).

The analysis also showed that M2-TAMs were the most abundant immune cells across the 22 analyzed cell types (Figure 6A, 6B). Furthermore, we analyzed six single-cell RNA-Seq data of PCa to characterize the cellular source of the CD274 expression across multiple cell types. Notably, the results suggested that Monocells/macrophages exhibit a higher expression of CD274 compared with other cells (Figure 6C–6E), suggesting TAMs were indeed the source of PD-L1 expression in bulk tumors. We also found that M2-TAMs were significantly negatively correlated with a large fraction of other immune cells, strongly suggesting that M2-TAMs play important roles in immune modulation in PCa (Figure 6F, 6G).

Together, these results suggest that M2-TAMs infiltration is significantly associated with PD-1 blockade therapy. The levels of regulatory T cells, memory T cells, memory B cells, plasma cells, as well as the M2-TAMs, may be involved in the modulation of the tumor microenvironment in PCa and can serve as targets for improving the efficacy of immunotherapy in the treatment of PCa.

## DISCUSSION

In the present study, we first examined CD68, CD163, and PD-L1 expression levels in PCa tissues. CD68 is a marker of M1- and M2-activated TAMs. We found that CD68 staining was weak to moderate in normal prostate tissue and tumor-adjacent cells but strong in PCa cells. This finding was similar to gastroesophageal adenocarcinoma, which displayed low-to-moderate CD68 expression in cancerous cells [26]. The IHC analysis of CD68+ TAM infiltration both in tumor and tumor-adjacent tissues showed a weak association with clinicopathological variables and BCR-free time, which may be a result of the limited sample size. However, in gastric cancer, CD68 was shown not to be associated with poor prognosis [27]. Conversely, Ilseon Hwang et al. reported that high CD68 expression in tumor stroma is associated with a good prognosis in non-small cell lung cancer (NSCLC) [19]. The high density of TAMs in colorectal cancer also seems to be associated with better survival, while no significant correlation was observed with survival in some esophageal cancer patients [5]. Cao et al. also found no correlation between CD68 density in the tumor interstitial region and overall survival (OS) of NSCLC patients [28]. In summary, the explanation for these inconsistencies may be related to the differences in tumor biology and unique characteristics of TAMs, which have dynamic and heterogeneous properties in response to certain local tumor microenvironments.

In the present study, we also examined CD163, a highly specific marker for M2-TAMs. Different from CD68, the number of CD163+ infiltrating TAMs, as well as CD68 mRNA expression, was increased with Gleason score and associated with risk stratification of PCa patients. A high level of CD68 mRNA/protein expression indicates a shorter BCR-free time. This was similar to a previous report by Marina Kazantseva and colleagues, which found that high levels of CD163+ TAM infiltration were associated with higher Gleason score [29]. Consistently, in cervical cancer and oral carcinomas, high levels of CD163+ TAMs infiltration were also associated with worse disease-free survival (DFS) [10, 30, 31].

Accumulated evidence suggests that a higher CD163+ TAMs count indicates poor prognosis and high metastatic potential in various cancers [32]; however, how CD163 works in the protumoral activation of TAMs remains unclear. Our data showed that the infiltration levels of M2-TAMs were significantly associated with Gleason score and the prognosis of PCa patients. Many studies have confirmed that M2-TAMs may directly communicate with cancer stem cells and promote their stemness and subsequent oncogenic properties, thereby triggering tumor invasion and metastasis [33]. Insulin-like growth factor (IGF) secreted by M2-TAMs has been shown to be involved in the occurrence and development of PCa, which activates the IRA/IGF1R-mediated PI3K/AKT/mTOR signaling pathway [34]. In summary, this evidence suggests that the subtype of infiltrated TAMs, especially the M2 subtype, could be an indicator for PCa progression and thus needs to be deeply explored.

PCa has been recognized as an immune “cold” tumor that is inert to common immunotherapy [35]. However, little knowledge is known about PD-L1 expression in PCa. Recent studies reported that CD163+ TAMs were associated with PD-L1 expression on tumor cells in several human cancers, including gastric adenocarcinoma, ovarian cancer, NSCLC, and so forth [36–39]. In good agreement, we found that M2-TAMs were positively associated with PD-L1 expression, and both of them were associated with the prognosis of PCa patients. In this work, we analyze the potential correlation between infiltrated TAMs and PD-L1 expression by IHC staining of TMAs and by CIBERSORTx analysis of RNA-Seq datasets. IHC results showed that 14.4% of PCa patients have high PD-L1 expression. Notably, levels of PD-L1 expression were positively correlated to Gleason score and its high expression is also associated with worse BCR-free survival, indicating that PD-L1 may be a co-factor associated with the progression of PCa, which is consistent with Juan He et al. and Gevensleben H et al.’s report [40, 41]. Interestingly, M2-TAMs infiltration was significantly positively correlated to PD-L1 expression in both local and public cohorts, suggesting that M2-TAMs infiltration could be used as a predictive marker for PD-L1 expression, which may link with immune escape in PCa. Tyro, Axl, and Mertk, collectively called TAM receptors, can activate the expression of PD-L1 in tumor cells, and additionally, IFN-γ secreted by inflammatory cells in the tumor microenvironment is associated with macrophage differentiation. Interestingly, IFN-γ induces the expression of PD-L1 in the tumor cells [42]. Fujita et al. and Wölfle S.J et al. reported that IL-8 produced by cancer cells stimulates CD163+ M2-TAMs to produce IL-10, which, in turn, leads to the phosphorylation of STAT3, and then IL-10/STAT3 signaling induces PD-L1 overexpression [43, 44]. We speculate that this relationship between M2-TAMs and PD-L1 expression could be the link between inflammation and immune escape in PCa, this speculation was further supported by the GSEA analysis. Therefore, exploring the strategies that target M2-TAMs and/or PD-L1-related signalings will shed new light on the management of late-stage PCa by improving the efficacy of ready-in-use immunotherapy.

One of the primary observations from previous research has been the propensity of M2-TAMs to facilitate tumor progression, angiogenesis, and metastasis. These macrophages, under the influence of tumor-derived factors, adopt an M2 phenotype, which is known for its pro-tumorigenic characteristics [45]. Our findings corroborate this understanding, but they also shine a light on a critical association with PD-L1 expression. Elevated PD-L1 expression in tumors has been extensively linked to immune escape mechanisms, leading to reduced T-cell mediated tumor elimination and thus, unfavorable clinical outcomes [39]. Given this background, our observations that CD163+ TAMs infiltration is significantly associated with the Gleason score and risk stratification of PCa adds another layer of understanding. The pronounced correlation between high infiltration of CD163+ TAMs and elevated PD-L1 expression suggests a potential synergy in driving immune escape mechanisms in PCa. This link could offer a nuanced understanding of why prostate cancer, traditionally considered a “cold” tumor, might be resisting immunotherapy modalities. Furthermore, our results emphasize that patients with high levels of CD163+ TAMs and PD-L1 expression have a truncated time to biochemical recurrence-free survival. This finding builds upon previous work1, underscoring the prognostic value of M2-TAMs and PD-L1 expression. It further hints at the possible therapeutic potential of targeting this axis for improved treatment outcomes. In light of the recent studies on checkpoint inhibition in prostate cancer [46], our study offers a fresh perspective. By illuminating the association between TAMs and PD-L1, we pave the way for more informed therapeutic strategies targeting this axis. However, it’s crucial to understand the exact molecular mechanisms underlying this association to design effective interventions.

While PCa is traditionally deemed an immune “cold” tumor, implying limited immune cell infiltration and immunotherapy responsiveness, our findings, alongside those from other studies, suggest the tumor microenvironment may be more nuanced than previously understood. The presence of M2-TAMs and their positive correlation with PD-L1 expression in PCa patients suggests that there is an active immunosuppressive environment present. In breast cancer, another traditionally recognized “cold” tumor, the role of macrophages and PD-L1 is also starting to be unveiled. Like PCa, M2-TAMs infiltration and elevated PD-L1 expression could be orchestrating an immune-evasive environment. The contribution of PD-L1 and macrophages to the immune escape mechanism could represent a common trait in these tumors, underscoring the significance of these findings in our study and their potential implications for other “cold” tumors. From a therapeutic standpoint, our findings might open up novel avenues in managing PCa. If M2-TAMs can be recognized as predictive markers for PD-L1 expression, strategies can be designed to target M2-TAMs either to repolarize them to a more anti-tumorigenic M1 phenotype or to reduce their recruitment to the tumor microenvironment This, in combination with PD-L1 inhibitors, could potentially remodel the immunosuppressive tumor microenvironment of PCa and convert it from “cold” to “hot,” enhancing its susceptibility to immunotherapies. Furthermore, the involvement of cytokines like IL-8 and IL-10 offers more potential therapeutic targets. Inhibiting these signaling pathways could disrupt the M2-TAM-mediated upregulation of PD-L1, potentially reducing immune evasion. In conclusion, the intricate interplay between M2-TAMs and PD-L1, and its consequential effect on the prognosis of PCa patients, presents an exciting frontier in the field of oncology. As more is discovered about these interactions, the possibility of transforming “cold” tumors like PCa and breast cancer into more immunotherapy-responsive malignancies becomes increasingly tangible. Our findings, when viewed in the context of the broader tumor microenvironment, underscore the importance of a multifaceted approach in harnessing the immune system against these traditionally challenging tumors.

A possible limitation of this study is that we used CD163 as a marker for M2-TAMs, which is the most currently used in the literature [22]; however, it is worth noting that M1- and M2-TAMs are the extremes of a continuous spectrum of macrophage polarization, and TAMs display high plasticity in response to different stimuli [47]. Consequently, some researchers consider that the single staining with CD163 is not sufficient for allocating macrophages towards M2 polarization [48]. Another potential limitation is that this is a retrospective study that cannot exclude potential selection bias. Thus, further molecular biology-based studies should be performed to confirm or extend the results. Besides, more efforts (such as single-cell transcriptomic analysis) may be needed to illustrate the potential role and relevant mechanisms among M1/M2-TAMs, and PD-L1 expression in the diagnosis, prognosis, and especially the treatment of PCa.

## CONCLUSIONS

In conclusion, our study demonstrates that tumor-associated macrophages (TAMs), particularly the M2 subtype, are increased in prostate cancer (PCa) and are associated with a poor prognosis for patients. The infiltration of both CD68+ and CD163+ TAMs was found to be significantly correlated with high PD-L1 expression in tumors, indicating a possible connection between TAM infiltration and immune escape in PCa. Developing therapeutic strategies that target TAMs, specifically, M2-TAMs, may enhance the efficacy of immunotherapy for PCa patients. This knowledge will be valuable for guiding future research on novel treatment approaches and improving patient outcomes in PCa.

## Supplementary Materials

Supplementary Figures

Supplementary Table 1

Supplementary Table 2
