# Supplementary material for: Tumor-associated macrophages and PD-L1 in prostate cancer: a possible key to unlocking immunotherapy efficacy
Source: Aging (Albany NY). 2024 Jan 4;16(1):445–65. doi: 10.18632/aging.205378 (PMC10817380; doi:10.18632/aging.205378)
Supplement: Supplementary Figures [file aging-16-205378-s001.pdf]

SUPPLEMENTARY FIGURES

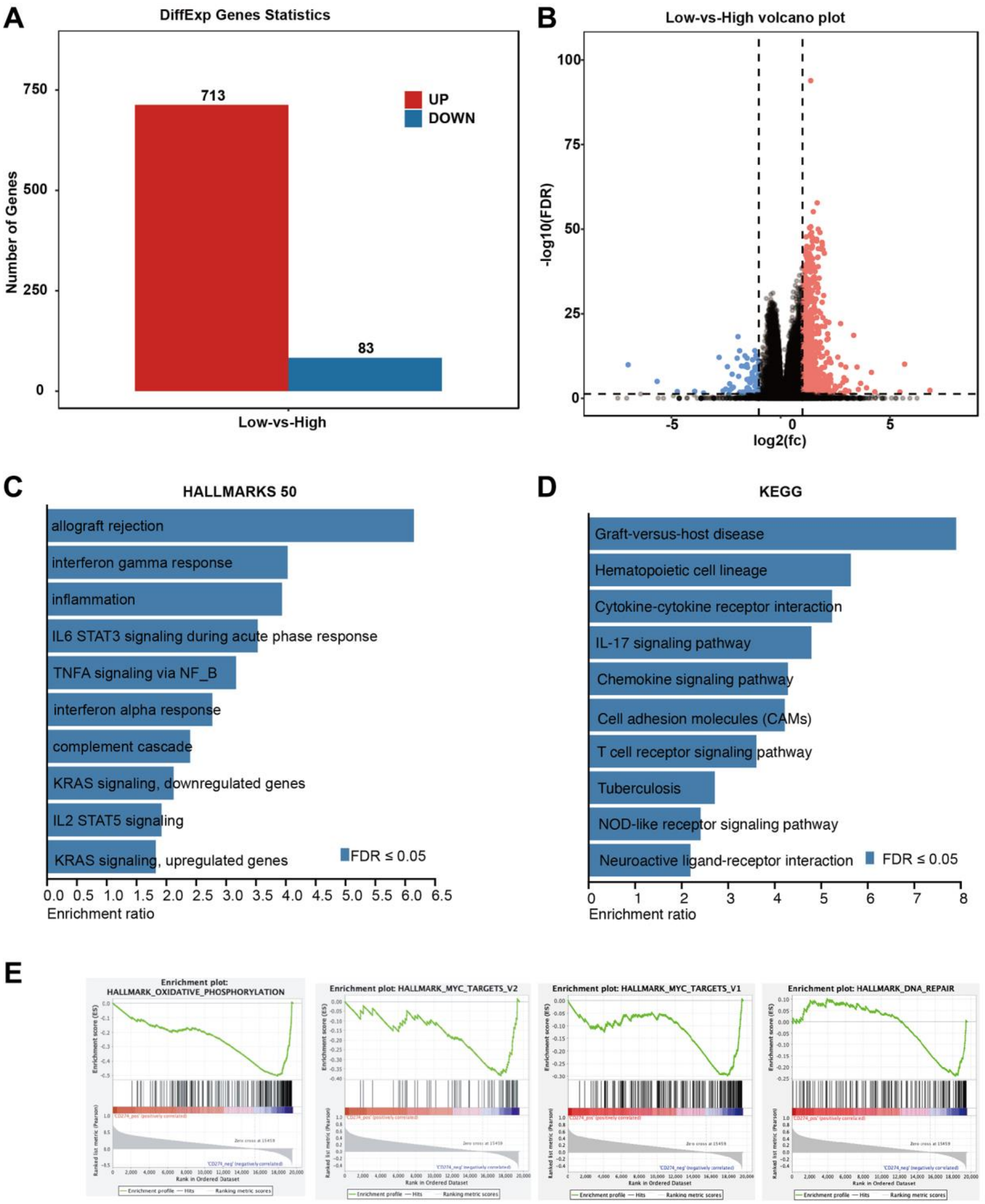

Supplementary Figure 1. PD-L1 expression was correlated with immune-related signaling in PCa. (**A**) Bar plot shows the number of differentially expressed genes between PCa samples with high and low PD-L1 expression. (**B**) Volcano plot shows the distribution of the differentially expressed genes. (**C**, **D**) Barplots show the over-representation analysis of the differentially expressed genes. (**E**) Gene set enrichment analyses show signaling pathways that are negatively related to PD-L1 expression.

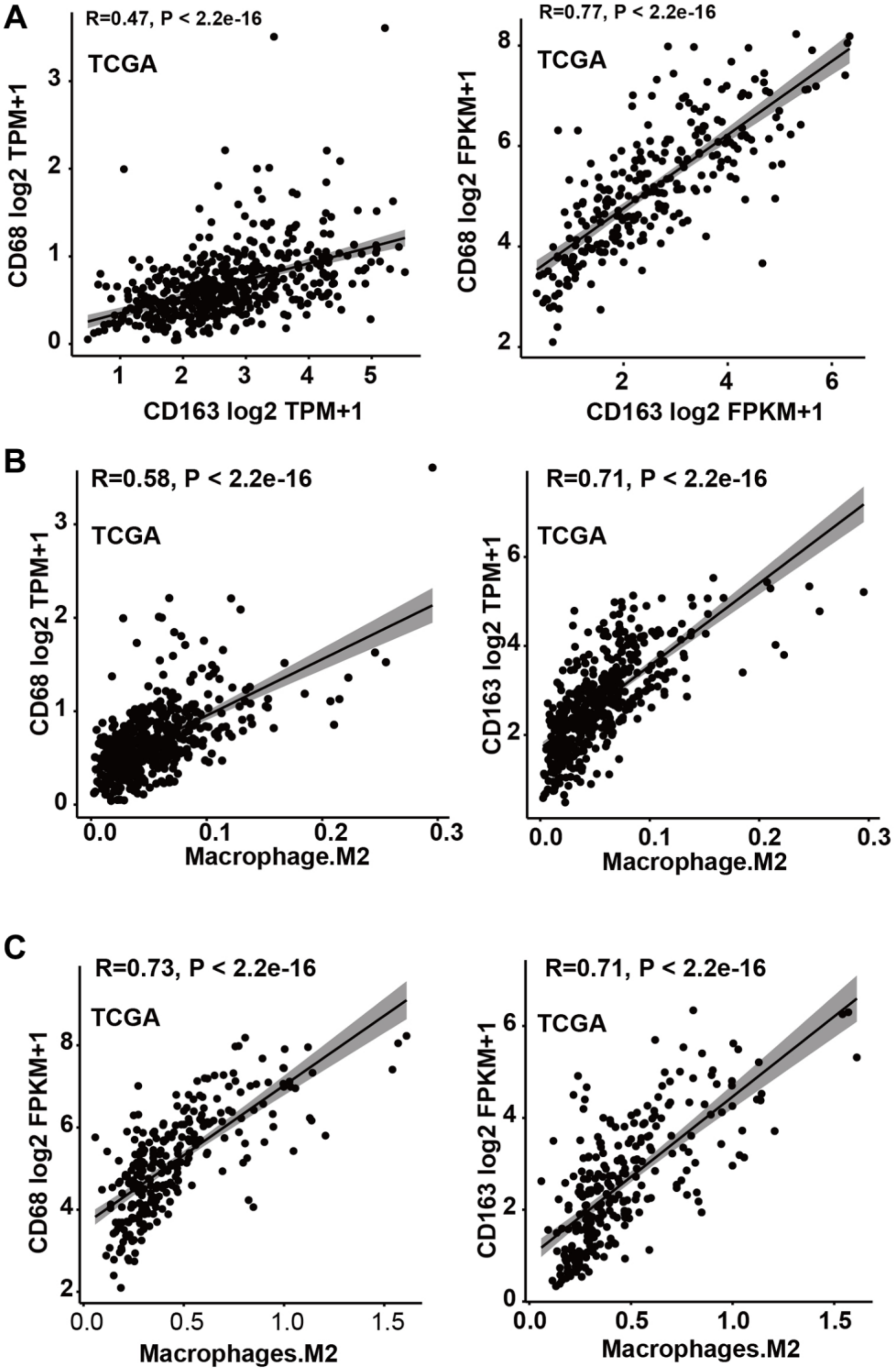

Supplementary Figure 2. Correlation among CD168, CD63, and M2-TAM in RNA-Seq datasets. (**A**) The correlation between the mRNA levels of CD68 and CD163. (**B**) Correlation between the infiltration levels of M2-TAMs and CD68 mRNA. (**C**) The correlation between CD63 mRNA and M2-TAMs infiltration.
